# Impact of temperature on postdiapause and diapause of the Asian gypsy moth, *Lymantria dispar asiatica*

**DOI:** 10.1093/jis/14.1.5

**Published:** 2014-01-01

**Authors:** Jing Wei, You-Qing Luo, Juan Shi, Dei-Peng Wang, Shao-Wei Shen

**Affiliations:** Forestry College, Beijing Forestry University, P.O. Box 113, Beijing 100083, China

**Keywords:** egg hatch, forest pest, gypsy moth distribution

## Abstract

*Lymantria dispar asiatica* (Vnukovskij) (Lepidoptera: Lymantridae) is one of three gypsy moth subspecies found in East Asia. Understanding the diapause and postdiapause phases of its eggs is important in characterizing its life cycle. The effects of different constant temperatures for different lengths of times on field-collected, postdiapause eggs were tested during the first year. In the second year, the effects of the same treatments on laboratory-raised eggs in diapause were investigated. The effects of temperature on percent egg hatching, time to hatching, and hatching duration were determined. When field-collected eggs were held at 0 and 5°C, they terminated postdiapause within 11 days. The percent hatching tended to decline with an increased duration of exposure at temperatures greater than 5°C. Diapause terminated slowly (> 37 days) and with a high percentage of hatching for postdiapause eggs held at 10°C. There was a positive correlation between temperature and the speed of postdiapause development for field-collected eggs held at constant temperatures between 10 and 25°C. However, the number of days to the first hatch was significantly longer than for eggs treated with lower temperatures before being transferred to 25°C. Freshly oviposited eggs treated at a constant 0 or 5°C for 200 days were unable to develop into pharate larva. However, eggs treated at a constant 20 or 25°C for 200 days developed into pharate larva but did not hatch even after a subsequent chill. This result suggests why *L. dispar asiatica* is not found in tropical areas and helps us to predict the distribution of the gypsy moth in China.

## Introduction

The gypsy moth, *Lymantria dispar* Linnaeus (Lepidoptera: Lymantridae), consists of three subspecies, the North American/European gypsy moth ( *L. dispar dispar* L), the Asian gypsy moth ( *L. dispar asiatica* Vnukovskij), and the Japanese gypsy moth ( *L. dispar japonica* Motschulsky) ( Pogue and Schaefer 2007 ). *Lymantria dispar* has become an important pest worldwide in temperate forests ( Davidson et al. 2001 ; Orozumbekov et al. 2009 ).

*L. dispar* is a univoltine species. Adult moths appear in late July or August. Eggs are laid soon after mating, an embryonic larva develops, and diapause occurs. Diapause allows the species to survive overwintering and enables its eggs to survive for up to 9 months, which accounts for 3/4 of the moth’s annual life ( Leonard 1968 ). Egg hatching is the most critical life-stage event in establishing seasonality of this pest (Gray 2003). These factors are important for the distribution of the *L. dispar* and have led to several studies focusing on the diapause mechanisms ( Bell 1996 ; Keena 1996 ; Gray et al. 2001 ).

Diapause is a dynamic process consisting of several distinct phases: induction, preparation, initiation, maintenance, termination, and postdiapause quiescence ( Kostál 2006 ). Three distinct phases: prediapause, diapause, and postdiapause have been noted in *L.dispar* egg development ( Sawyer et al. 1993 ). In the prediapause phase, embryos begin development with high respiration rates, with the developmental rate favored by a high temperature (30°C) ( Gray et al. 1991 ), that promotes morphological development ( Leonard 1968 ). They then enter diapause as fully differentiated pharate larvae ( Bell 1996 ). During diapause, the respiration rate is low, the developmental rate is favored by low temperatures (5°C), and morphological development does not occur ( Gray et al. 2001 ). In the postdiapause phase, respiration rates are again high, and the developmental rate is again favored by high temperatures ( Gray et al. 1995 ). After the completion of postdiapause, eggs begin to hatch.

Temperature is a key factor in terminating diapause and postdiapause in *L. dispar* . In many models that predict the invasive potential of gypsy moths, temperature is considered to be the most important factor ( Gray 2009 ; Pitt et al. 2007 ; Régnière et al. 2009 ). Tauber et al. (1990) indicated that temperature plays a large role in diapause maintenance and the timing of postdiapause egg hatching. They hypothesized a model of egg development encompassing the diapause and postdiapause phases. Diapause development is considered a dynamic process in which the egg’s response to temperature changes gradually during the course of development. However, there is no clear dividing line between the diapause and postdiapause phases. At the start of postdiapause, eggs are sensitive to temperature variation, which allows them to withstand harsh conditions while being ready to hatch quickly when favorable environmental conditions occur ( Gray et al. 1991 ; Gray et al. 1995 ). Diapause was gradually terminated by chilling the eggs for several months, but eggs of different ages had different responses to the chill duration ( Bell 1996 ). The exposure of eggs to different durations of different low temperatures showed that hatching takes longer to begin, and proceeds more slowly, in eggs held at 15 or 20°C and that more than 99% of the eggs held at 25°C did not hatch ( Keena 1996 ). In this experiment the eggs were transferred from 5°C to different temperatures for varying periods of time. Eggs held at 25°C maintained the longest diapause periods, and 75 days at 5°C followed by a switch to 25°C resulted in the shortest diapause period. A longer duration at 5°C resulted in a shorter diapause period ( Gray et al. 2001 ).

Gray and Keena (2005) compared diapause in *L. d. asiatica* and *L. d. dispar* strains under different temperature regimes and found that *L. d. asiatica* completed diapause faster than *L. d. dispar* . However, whether the postdiapause of *L. dispar asiatica* collected from Chinese fields is different from that of *L. d. dispar* has not been sufficiently examined. Therefore, *L. d. asiatica* populations from different regions of China were evaluated to assess their general postdiapause characteristics. The impact of temperature on the postdiapause and diapause phases of *L. d. asiatica* was tested by using different time periods of constant temperatures. The effects of various temperatures on diapause are discussed, which will help to predict the potential distribution of *L. d. asiatica* in China.

## Materials and Methods

### Gypsy moth eggs

In 2010 *, L. d. asiatica* egg masses (> 50) were collected from De Lisi in the Liao Ning province (LN strain, 122°04'E, 39°47'N, average January and July temperatures of -4.9 and 23°C, respectively), Zhang Jiakou in the He Bei province (HB strain, 114°52'E, 40°49'N, average January and July temperatures of -9.7 and 23.2°C, respectively), and the Fang Shan zone of Beijing (BJ strain, 116°8'E, 39°44'N, average January and July temperatures of -4.6 and 25.8°C, respectively), all in China. Egg masses were primarily collected from the eaves of houses made of soil near larch ( *Larix* ) or poplar ( *Populus* ) forests on February 1st (LN strain) and February 6th (HB and BJ strains). In 2011, egg masses were collected from the tree bark of larch ( *Larix* ) at Aer Shan in Inner Mongolia (IM strain, 119°56'E, 47°10'N, average January and July temperatures of -25.6 and 16.6°C, respectively) on January 21st. All egg masses were sent to Beijing within two days in a plastic box inside a dark paper box. They were disinfected with 1% formaldehyde immediately after arrival. Each egg mass was placed in 1% formaldehyde for 3 seconds and taken out for 15 seconds; this procedure was repeated three times for each egg mass. The eggs were immediately subjected to the designed temperature regimes. The climate data of each site were determined using the Thermatic Hu-man-Earth System ( http://www.data.ac.cn/ ).

### Experimental treatments

**Experiment 1.** The impact of different temperatures and durations on eggs was tested in an experiment set up in a two factor, completely randomized design. The temperatures were 0, 5, 10, 15, and 20°C. The times were 0, 30, 60, and 90 days. Egg masses were randomly assigned to different treatments and then transferred to a constant 25°C incubator (16:8 L:D, RH 60–70%, Model: Safe PGX-250A, H2O3=100 c/75c). Each combination included three replicates of 50–100 eggs.

**Experiment 2.** The effect of low temperature (5ºC) on postdiapause was evaluated in a single factor, completely randomized design. Field-collected eggs of the LN strain were held at 5°C for 60 days and then randomly transferred to 0, 10, 15, 20, or 25°C. Each treatment utilized three replicates of 50–100 eggs.

**Experiment 3.** Based on previous work, an experiment was designed to more accurately assess the impact of temperature on diapause and postdiapause. A quadratic regression orthogonal design was applied to test the impact of postdiapause temperatures of 5, 6.3, 15, 23.7, and 25°C for durations of 0, 5.8, 45, 84.1, and 90 days. Eggs from IM (2011) were held at 5°C for different durations and then transferred to different temperature regimes. Each treatment included 30–50 eggs. In 2010, *L. d. asiatica* eggs were collected from three places (LN, BJ, and HB) and raised in the laboratory. Adults were randomly mated within the population, and egg masses were collected. These egg masses were subjected to 0, 5, 20, and 25°C within 2 days of being laid. Two hundred days later, when the IM strain was collected in 2011, the eggs were transferred to the quadratic regression orthogonal design experiment. A small portion of eggs from the three populations (BJ, LN, and HB) were stored under constant temperatures of 0, 5, 20, and 25°C. Eggs collected from the BJ population in 2010 were stored at temperatures of 0 and 5°C. These eggs were moved to this experiment in 2011 as well. These tests were done to determine if holding eggs at 0 or 5°C prolongs postdiapause. Each combination included three replicates of 50–100 eggs.

### Statistical analysis

The percent egg hatching (larvae successfully emerging from an egg mass), time to hatching (time from the day the eggs were subjected to different temperatures until the larvae started to hatch), and hatch duration (time from the first to last hatchings) were noted, and the percent hatching was transformed to arcsine (square root). Treatment time, temperature, and interactions were analyzed using a two-way ANOVA ( R Core Team 2013 ). Multiple comparison testing was performed using Tukey’s HSD, using the R agricolae packages ( de Mendiburu 2013 ). Means were separated as significant using an ANOVA at *p* < 0.05. Quadratic regression orthogonal design was analyzed as noted in Xu and Huang (1995) . The figure was created using Origin 8.1 software (OriginLab Corporation, http://www.originlab.com/ ).

## Results

### Experiment 1

**Figure 1.**
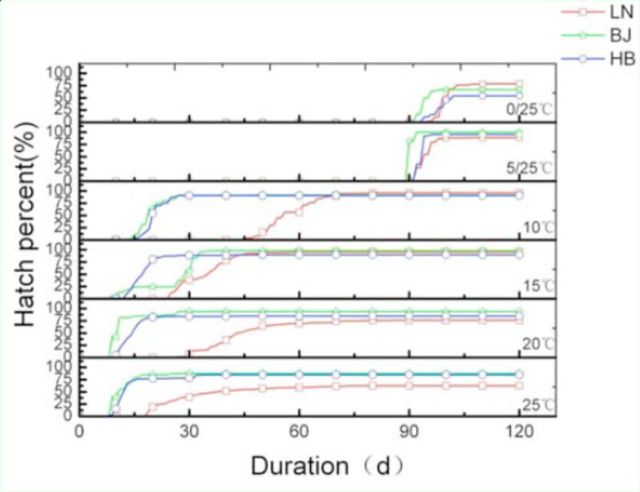
Cumulative percentage hatching of embryonated eggs for gypsy moth populations from LN (red line with squares), BJ (green line with stars), and HB (blue line with circles). The notation 0/25°C and 5/25°C indicates 90 days at 0 or 5°C and then a transfer to 25°C. No larvae emerged before being switched to 25°C. The 10, 15, and 20°C notation indicates that eggs were treated constantly at those temperatures. Those eggs hatched without being transferred to 25°C. BJ, Beijing strain; HB, He Bei strain; IM, inner Mongolian strain; LN, Liao Ning strain. High quality figures are available online.

For all three populations, field-collected eggs held at 0°C and 5°C started to hatch after being transferred to 25°C. A portion of eggs held at 10, 15, and 20°C began to hatch before being moved to 25°C ( Figure 1 ).

### Percent egg hatching

For the LN strain, the percent hatching at 0 days was significantly lower than for the other treatments ( *F* = 76.74; df = 3, 60; *p* < 0.01, Table 1 ). At 10°C, the percent hatching was significantly higher than at 25°C ( *F* = 11.20; df = 4, 60; *p* < 0.01, Table 2 ), and the interaction was also highly significant ( *F* = 3.46; df = 12, 60; *p* < 0.01). For the BJ strain, the percent hatching rates between treatment times were significantly different by ANOVA ( *F* = 3.10; df = 3, 60; *p* = 0.0373) but not with Tukey’s HSD multiple comparison (hereafter referred to as Tukey’s). The percent egg hatching at 20°C was significantly higher than at 0/25°C ( *F* = 3.36; df = 4, 60; *p* = 0.0183, Table 2 ), and the interaction was not significant ( *F* = 1.18; df = 12, 60; *p* = 0.3241). Using the HB strain, the percent hatching at 0 days was higher than at 90 days ( *F* =4.11; df = 3, 60; *p* = 0.0124, Table 1 ), but it did not significantly depend on the treatment ( *F* = 0.87; df = 4, 60; *p* = 0.4862, Table 2 ). The interaction was also not significant ( *F* = 1.08; df = 12, 60; *p* = 0.3945). These results are summarized in Figure 1 .

**Table 1.**
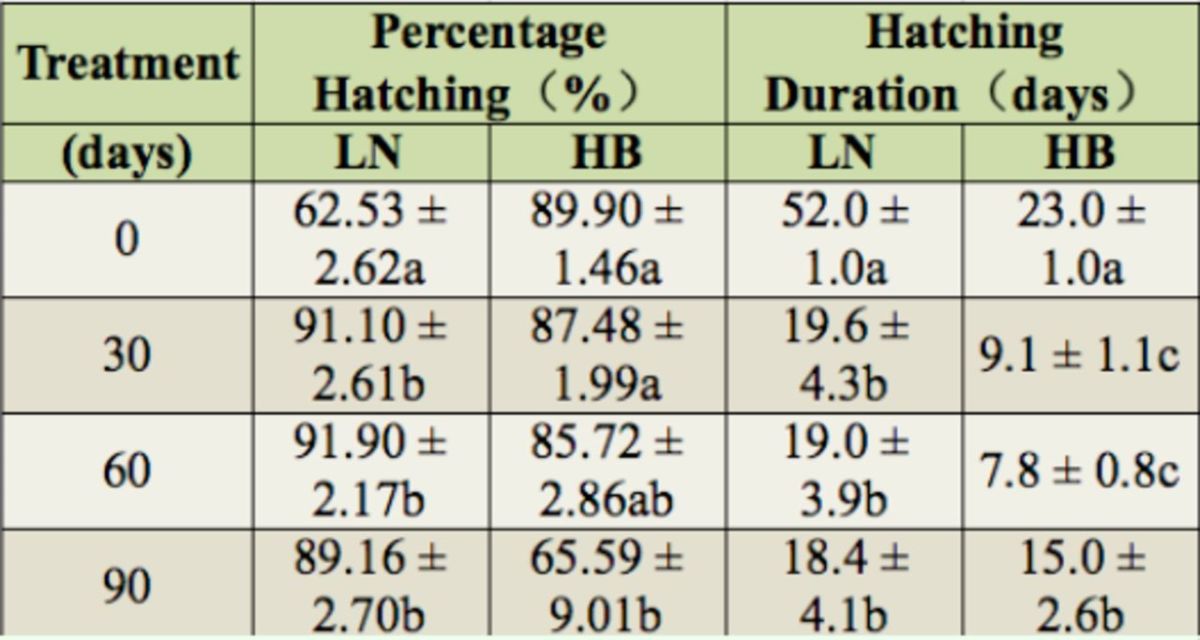
Comparison of the mean percent hatching and hatching duration of the LN and HB populations in 2010 treated for different durations. Means followed by the same letter are not significantly different ( *p* < 0.05, mean ± SE). Abbreviations as in Figure 1 .

**Table 2.**
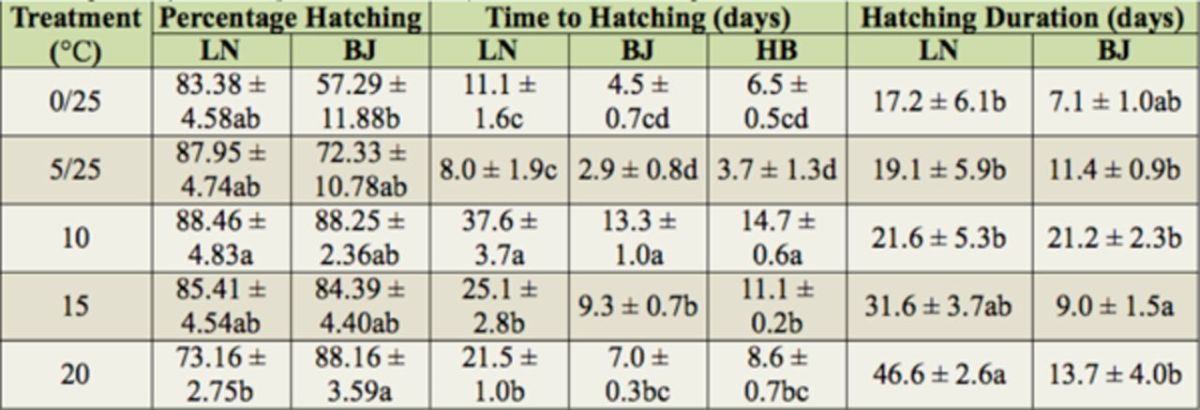
Comparison of the hatching of the LN and BJ populations, the time to hatching of the LN, BJ, and HB populations, and the hatching duration of the LN and BJ populations in 2010 treated at different temperatures. Means followed by the same letter are not significantly different ( *p* < 0.05, mean ± SE). Abbreviations as in Figure 1 .

### Time to hatching

The LN population times to hatching after various treatment times were significantly different by ANOVA ( *F* =3.98; df = 3, 60; *p* = 0.0141) but not using Tukey’s. The time to hatching at 10°C was greater than that for the other treatments ( *F* = 73.27; df = 4, 60; *p* < 0.01, Table 2 ), and the interaction was significant ( *F* = 9.83; df = 12, 60; *p* < 0.01). With the HB strain, the times to hatching after different treatments were also significantly different under ANOVA ( *F* = 4.42; df = 3, 60; *p* = 0.00885) but not by Tukey’s. Like the LN strain, the time to hatching at 10°C was significantly higher than that for the other temperatures ( *F* = 81.19; df = 4, 60; *p* < 0.01, Table 2 ), and the interaction was significant ( *F* = 9.28; df = 12, 60; *p* < 0.01). With the BJ strain, unlike the two previous strains, the times to hatching were not significantly different between the treatment times ( *F* = 2.69; df = 3, 60; *p* = 0.0587). However, as with the two other strains, the time to hatching at 10°C was significantly higher than those of the other treatments ( *F* = 98.14; df = 4, 60; *p* < 0.01, Table 2 ), and the interaction was significant ( *F* =12.57; df = 12, 60; *p* < 0.01).

### Hatching duration

The LN hatching duration at 0 days was significantly higher than those of the other treatments ( *F* = 140.16; df = 3, 60; *p* < 0.01, Table 1 ). At 20°C, it was significantly higher than at 0/25, 5/25, and 10°C ( *F* = 61.17; df = 4, 60; *p* < 0.01, Table 2 ), and the interaction was also highly significant ( *F* = 7.68; df = 12, 60; *p* < 0.01). For HB eggs, the hatching duration at 0 days was significantly higher than those of the other treatments ( *F* = 37.42; df = 3, 60; *p* < 0.01, Table 1 ). Although the hatching durations between treatment temperatures were highly significantly different with ANOVA ( *F* = 7.37; df = 4, 60; *p* = 0.000152), they were not significantly different using Tukey’s. However, the interaction was significant ( *F* = 2.45; df = 12, 60; *p* = 0.016862). The BJ strain hatching duration was significantly different between treatment times with ANOVA ( *F* = 8.10; df = 3, 60; *p* = 0.000247) but not with Tukey’s. The hatching duration at 10°C was higher than at 5/25, 10, and 20°C ( *F* = 22.15; df = 4, 60; *p* < 0.01, Table 2 ), and the interaction was significant ( *F* = 12.35; df = 12, 60; *p* < 0.01).

### Experiment 2

The eggs did not hatch when transferred to 0ºC and held at 5ºC. The hatching rates of the eggs transferred to 10, 15, 20, and 25ºC were not significantly different ( *F* = 110.5; df = 5, 12; *p* < 0.01, Table 3 ). The eggs transferred to 20ºC and 25ºC had shorter times to hatching than eggs transferred to 10ºC ( *F* = 7.26; df = 3, 4; *p* = 0.0113, Table 3 ). There were no significant differences in hatching duration when comparing the temperatures to which the eggs were transferred ( *F* = 1.86; df = 3, 4; *p* = 0.214).

**Table 3.**
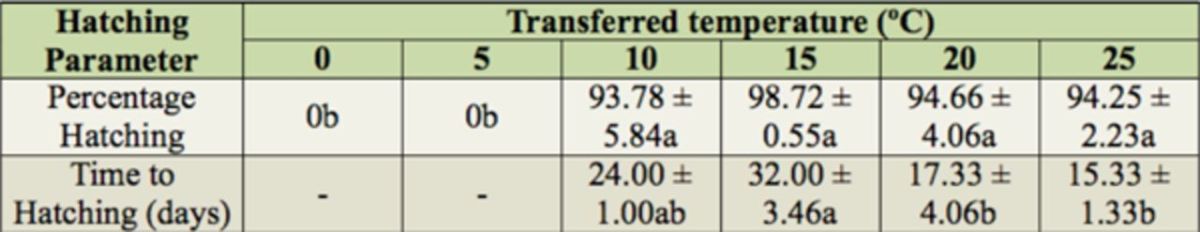
Percentage hatching and time to hatching of eggs of the LN population held at 5ºC for 60 days and then transferred to five different constant temperatures. Means followed by the same letter are not significantly different ( *p* < 0.05, mean ± SE). LN, Liao Ning strain.

### Experiment 3

**Effects of temperature on prediapause development and hatching.** Fewer than 0.001% of laboratory-raised eggs from the three populations (LN, BJ, and HB) hatched. Eggs held at constant temperatures of 0, 5, 20, and 25°C did not hatch. Eggs originally held at a constant 20 or 25°C developed into fully differentiated pharate larva, but those held at a constant 0 or 5°C were undeveloped. Moreover, BJ populations collected in 2010 and held at a constant 5°C did not hatch after being moved to higher temperatures in 2011.

**IM population** . Egg hatching for the Inner Mongolia strain was gradual. The quadratic regression equation for the relationship between treatment time and temperature verses percentage hatching was calculated as follows:

$Y = -0.039x_{1}^{2} - 0.184x_{2}^{2} + 0.34x_{1}x_{2}-0.449x_{1} + 0.122x_{2} + 0.535$

where Y is the hatching percentage, x _1_ is the treatment time, and x _2_ is the treatment temperature. R ^2^ = 0.874 and *p* < 0.05.

The quadratic regression equation between treatment time and temperature to the time to hatching was as follows:

$Y = 5.388x_{1}^{2} + 1.594x_{2}^{2} + 6.418x_{1}x_{2}-6.849x_{1}-2.618x_{2} + 5.241$

where Y is the time to hatching, x _1_ is the treatment time, and x _2_ is the treatment temperature. *R*^2^ =0.871 and *p* < 0.05.

When postdiapause eggs were held at 5°C for 4.5–78.6 days and then moved to 7.16– 22.84°C, the egg hatching rate was greater than 70% with a confidence level of 95%. When postdiapause eggs were held at 5°C for 2.6–87.4 days and then moved to 8.72– 21.29°C, the time to hatching was greater than 20 days with a 95% confidence level. When the time at 5°C was increased, the percent hatching declined significantly.

## Discussion

To study the percent hatching, experiments on the time to hatching and the hatching duration of *L. dispar asiatica* were conducted at different temperatures and times. The results indicated that 0 or 5°C could stimulate field-collected eggs to break postdiapause more quickly. However, if the low temperature duration was too long, the percent hatching declined, which supports the observations by Keena (1996) and Gray and Keena (2005) . Keena (1996) pointed out that with strains from Massachusetts, USA and Russia, the longer the eggs were treated at 5°C, the less time it took them to hatch.

Here, eggs had a higher percent hatching at a constant 10 or 15°C but were slow to break postdiapause. Postdiapause eggs held at a constant 25°C had a lower percent hatching and longer hatching duration. This result likely occurred because the eggs had not received enough of a chill in the field before collection, which would have synchronized hatching. This result also is consistent with the findings of Gray and Keena (1995) . Keena (1996) studied the impact of temperature on diapause and postdiapause in *L. d. dispar* and *L. d. asiatica* strains. She indicated that after treatment at 5°C, when the eggs were transferred to suitable (higher) temperatures, postdiapause was effectively broken. That finding was also supported here, and our study revealed that low temperature could stimulate *L. dispar asiatica* to terminate postdiapause. Eggs treated for 30 days at 5°C and then transferred to 10°C, maintained a high hatching rate. However, that rate tended to decline with longer durations at low temperatures. Freshly laid eggs of the *L. dispar asiatica* Chinese population did not hatch after constant treatment at 0, 5, 20, or 25°C for 200 days. Eggs treated at 20 or 25°C developed into fully differentiated pharate larva, while those treated at 0 or 5°C were still in the yolk stage. This result suggested that eggs can develop into pharate larva and enter diapause under a constant temperature of 20 or 25°C. However, a constant 20 or 25°C temperature reduced the responses of the eggs to low temperature, so they did not terminate postdiapause. When the eggs were dissected, the egg yolks were depleted, suggesting that a low supply of yolk was another reason for the death of the larvae in the eggs. These results imply that *L. d. asiatica* is not able to finish their life cycles in areas without a cold winter. For example, Hai Kou (110°10′–110°41′E, 19°32′–20°05′N) has average January and July temperatures of 17.2°C and 28.4°C, respectively. *L. d. asiatica* eggs can develop into pharate larva in Hai Kou but do not break diapause due to a lack of low temperature stimulation.

Winter is cold enough in Northern China for gypsy moth eggs to break diapause and hatch, which explains the wide distribution of *L. dispar asiatica* in Northern China. On the other hand, several provinces in Southern China are in tropical or subtropical climate zones. In the Hai Nan, Guang Dong, and Guang Xi provinces, and in the southern parts of the Fu Jian province, the average temperature in winter is above 15°C, which may not provide enough chill for gypsy moth eggs to break diapause and proceed to hatch. More field investigations should be conducted to obtain detailed distribution information on *L. dispar asiatica* in China.

## Abbreviations

BJ: Beijing strain
HB: He Bei strain
IM: inner Mongolian strain
LN: Liao Ning strain
